# Evaluation of Dry Feed Formulations for Culturing the Commercial Fairy Shrimp *Streptocephalus sirindhornae*

**DOI:** 10.3390/biology15110893

**Published:** 2026-06-05

**Authors:** Kosit Sriphuthorn, Prapatsorn Dabseepai, Naiyana Senasri

**Affiliations:** 1 Department of Fisheries, Faculty of Natural Resources, Rajamangala University of Technology Isan Sakon Nakhon Campus, Sakon Nakhon 47160, Thailand; kosit.sr@rmuti.ac.th; 2 Applied Taxonomic Research Center, Department of Biology, Faculty of Science, Khon Kaen University, Khon Kaen 40002, Thailand; prapda@kku.ac.th

**Keywords:** aquaculture, live feed, commercial shrimp feed, spirulina powder, rice bran

## Abstract

This study evaluated locally available dry feed formulations (FFs) as practical alternatives to fresh *Chlorella* sp. for culturing the freshwater fairy shrimp *Streptocephalus sirindhornae*. Seven dietary treatments were evaluated, including fresh *Chlorella* sp. at 1 × 10^6^ cells mL^−1^ (FF1; control) and six mixed dry diets (FF2–FF7) formulated from spirulina powder, commercial shrimp feed, fish meal, and rice bran. Fairy shrimp were cultured for 20 days in a completely randomized design with three replicates per treatment at a stocking density of 30 individuals L^−1^. Growth performance (body length and wet body weight) and survival were assessed across three developmental stages (1–5, 6–10, and 11–20 days post-hatch). Across all developmental stages, FF2 (50% spirulina powder + 50% commercial shrimp feed) consistently supported culture performance comparable to that of the control treatment. During the early developmental stage (1–5 days post-hatch), shrimp fed FF2 exhibited growth and survival rates comparable to those of the control group and significantly higher (*p* < 0.05) than those observed in several other dry diet treatments. During the late developmental stage (11–20 days post-hatch), survival of shrimp fed FF2 (62.45 ± 5.28 percent) did not differ significantly from that of the control group (61.85 ± 4.25 percent) but was significantly higher (*p* < 0.05) than survival in the other dry diet treatments. In addition, shrimp biomass produced with FF2 showed greater protein, lipid, carotenoid, and amino acid contents than shrimp fed fresh *Chlorella* sp. Protein, lipid, and amino acid contents were determined using standard AOAC methods, and carotenoid content was analyzed by HPLC. These findings suggest that FF2 may serve as a practical algae-independent diet for maintaining growth and survival of *S. sirindhornae* under controlled hatchery conditions.

## Simple Summary

Fairy shrimp (*Streptocephalus sirindhornae*) are widely used as live feed in freshwater aquaculture. However, nursery culture of this species commonly relies on fresh microalgae, particularly *Chlorella* sp., which require continuous production and technical management. These constraints increase operational complexity and may reduce culture consistency. Therefore, identifying practical alternatives to live microalgae is important for improving hatchery sustainability. This study evaluated locally available dry feed formulations as alternatives to fresh *Chlorella* sp. during a 20-day nursery culture period. Growth performance, survival, and nutritional composition were compared among dietary treatments. A mixed diet consisting of equal proportions of spirulina powder and commercial shrimp feed consistently supported growth and survival rates comparable to those obtained with fresh microalgae and significantly (*p* < 0.05) higher than those observed in several other dry feed treatments. In addition, shrimp fed this mixed diet showed greater protein, lipid, and carotenoid contents than shrimp fed fresh *Chlorella* sp. These findings suggest that a spirulina–shrimp feed mixture may serve as a practical algae-independent feeding strategy for nursery culture of *S. sirindhornae* under controlled hatchery conditions.

## 1. Introduction

Ephemeral aquatic habitats are characteristically inhabited by anostracans (Branchiopoda), commonly known as fairy shrimp. These organisms occur widely in temporary freshwater and inland saline lentic ecosystems and exhibit pronounced physiological and ecological adaptations to highly unpredictable hydroperiods [1,2,3]. A key adaptive trait is the production of desiccation-resistant resting eggs (cysts) that can remain viable for years under dry conditions [4,5]. Following rainfall and inundation, cysts rapidly hatch (within 1–4 days), allowing populations to complete their life cycle within the limited duration of temporary ponds [6]. Tropical freshwater fairy shrimp have gained increasing attention in aquaculture as alternative live feed organisms owing to their rapid growth, short life span, high fecundity, and capacity to generate nutritionally valuable biomass [7,8,9,10]. Both live and processed fairy shrimp have been used successfully as feed for freshwater aquaculture species, including shrimp, prawns, and ornamental fish. Their high protein content, favorable amino acid composition, and carotenoid richness have been associated with improved growth, survival, and pigmentation of cultured animals [11,12,13,14,15]. Accordingly, tropical fairy shrimp have been proposed as promising alternatives to *Artemia* spp., particularly in freshwater hatcheries where *Artemia* cysts are costly, inconsistently available, or less suitable for low-salinity systems [14,16].

In Thailand, commercial fairy shrimp culture has primarily focused on *Streptocephalus sirindhornae* and *Branchinella thailandensis* since the early 2000s [16,17]. These species have been applied as live or dried feeds to enhance growth and carotenoid deposition in giant freshwater prawns (*Macrobrachium rosenbergii*) and ornamental fish, including flowerhorn cichlids [9,18,19]. Notably, *S. sirindhornae* nauplii supported higher specific growth rates in *M. rosenbergii* postlarvae than *Artemia* nauplii, *Moina*, or commercial feeds [18]. Related approaches have also been explored elsewhere, such as the mass culture of *Branchinecta orientalis* using trout pond effluent in recirculating systems [20]. Despite progress in culture systems, fairy shrimp production in Thailand has traditionally relied on fresh microalgae, particularly *Chlorella* spp., as the principal food source in laboratory systems, concrete hatcheries, and earthen ponds [6,9,21,22]. *Chlorella vulgaris* is a unicellular green microalga with a small cell size (2–10 µm), high digestibility, and an advantageous nutrient profile, making it suitable for early developmental stages [5,23,24]. Other microalgae (e.g., *Chlorococcum humicola* and *Pediastrum boryanum*) and photosynthetic bacteria (*Rhodopseudomonas faecalis*) have also been evaluated as alternative feeds with variable outcomes [16,22,25]. However, live algal production is often labor-intensive, environmentally dependent, and difficult to maintain consistently, particularly during the rainy season when reduced light availability limits photosynthesis. Unstable algal production can result in inconsistent feed availability, which may lead to inadequate or irregular nutrient intake by cultured organisms. In addition, poor control of algal density and quality may contribute to organic matter accumulation and deterioration of water quality, ultimately increasing the risk of culture failure [8,26]. These constraints have prompted interest in algae-independent feeding strategies based on dried or formulated feeds that can be stored, standardized, and applied more reliably at nursery scale. Among dried feed alternatives, *Spirulina platensis* has received considerable attention because of its high-quality protein content, carotenoids, antioxidant compounds, and widespread commercial availability [27]. In contrast, dried *Chlorella* has shown limited effectiveness in sustaining fairy shrimp survival beyond the early developmental stages [22]. However, no single feed ingredient is likely to provide all nutritional requirements required throughout the entire life cycle of fairy shrimp. Therefore, combining nutritionally complementary ingredients may improve dietary balance and feeding performance. These findings suggest that mixed feed formulations combining algal and non-algal ingredients may better satisfy the nutritional requirements of fairy shrimp across different developmental stages [12,19].

Therefore, the present study evaluated mixed dried feed formulations composed of locally available ingredients as alternatives to fresh *Chlorella* sp. for the culture of *S. sirindhornae*. Specifically, the objectives were to (i) compare the effects of six mixed dried diets and fresh *Chlorella* sp. on growth performance and survival across three developmental stages (1–5, 6–10, and 11–20 days post-hatch); (ii) assess water quality stability under different feeding regimes; and (iii) characterize the nutritional quality of shrimp biomass produced with the best-performing dried diet in terms of proximate composition, carotenoid content, and amino acid profile. The findings are expected to support practical, nutritionally adequate, and scalable feeding strategies that reduce dependence on live microalgae in freshwater fairy shrimp culture.

## 2. Materials and Methods

### 2.1. Experimental Diets

Six dried feed formulations were evaluated in this study, with fresh *Chlorella* sp. used as the control diet. Stock cultures of *Chlorella* sp. were obtained from the Applied Taxonomic Research Center, Khon Kaen University, Thailand. Algae were cultivated in outdoor tanks under continuous aeration using a fertilization medium consisting of N–P–K fertilizer (16–20–0; 150 g), urea (46–0–0; 300 g), lime (CaCO_3_; 90 g), and rice bran (500 g) per cubic meter of water. After 3–5 days of cultivation, algal biomass was harvested and supplied to fairy shrimp at a concentration of 1 × 10^6^ cells mL^−1^, following Saengphan et al. [8].

**Figure 1:**
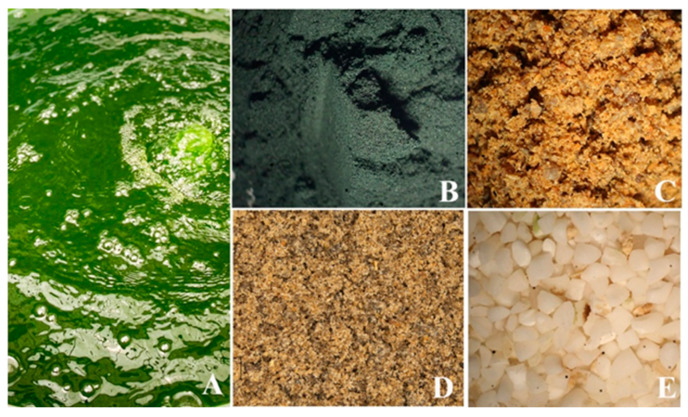
Experimental culture system and dry feed ingredients used in this study. Algae (*Chlorella* sp.) were cultivated in outdoor tanks (**A**). The dry feed ingredients consisted of spirulina powder (**B**), commercial complete shrimp feed (**C**), fish meal (**D**), and fine rice bran (**E**).

Four dried feed ingredients—spirulina powder (*Spirulina platensis*), commercial complete shrimp feed (Charoen Pokphand Foods Public Company Limited, Thailand; approximately 40% crude protein, 5% crude lipid, 4% crude fiber, and 12% ash according to the manufacturer’s specifications), commercial fish meal (approximately 50–65% crude protein), and fine rice bran—were purchased from local markets in Sakon Nakhon Province, Thailand. Fresh *Chlorella* sp. at 1 × 10^6^ cells mL^−1^ served as the control treatment (FF1). Six experimental dried diets (FF2–FF7) were prepared by mixing two ingredients at a ratio of 50:50 (*w*/*w*) as follows: FF2 (spirulina powder + complete shrimp feed), FF3 (spirulina powder + fish meal), FF4 (spirulina powder + fine rice bran), FF5 (complete shrimp feed + fish meal), FF6 (complete shrimp feed + fine rice bran), and FF7 (fish meal+ fine rice bran). All ingredients were finely ground and passed through a 250-µm sieve prior to mixing to improve suitability for early-stage fairy shrimp, particularly nauplii. The prepared diets were thoroughly mixed and stored in airtight containers at room temperature (28–30 °C) until use (Figure 1).

### 2.2. Experimental Design and Cultivation of Fairy Shrimp

All experimental procedures were conducted in accordance with the Thai Law on Animals for Scientific Purposes (2015) and approved by the Animal Ethics Committee of Rajamangala University of Technology Isan, Thailand (approval code: ID# U1-01774-2558). The experiment followed a completely randomized design (CRD) with seven dietary treatments (FF1–FF7) and three replicates per treatment. Fairy shrimp (*S. sirindhornae*) were cultured across three developmental stages: 1–5, 6–10, and 11–20 days post-hatch. For each stage, shrimp aged 1, 6, or 11 days post-hatch were stocked at a density of 30 individuals L^−1^ in 5-L circular plastic containers following [28]. Each container represented one experimental unit.

Resting eggs were obtained from the Faculty of Natural Resources, Rajamangala University of Technology Isan. Eggs were hatched in 1-L plastic containers under continuous aeration at room temperature (28–30 °C) using dechlorinated freshwater (0 ppt salinity) under natural photoperiod conditions. Approximately 1000 nauplii were transferred to 500-L fiberglass tanks containing 100 L of dechlorinated tap water, corresponding to an initial stocking density of approximately 10 nauplii L^−1^, and reared under continuous aeration. The nauplii were fed fresh *Chlorella* sp. twice daily at a concentration of 1 × 10^6^ cells mL^−1^ following the nursery culture protocol described by Saengphan et al. [8]. Shrimp of appropriate ages were randomly selected for feeding trials. Continuous aeration was provided throughout the culture period, and 10% of the culture water was exchanged daily using pre-aerated dechlorinated tap water maintained at the same temperature as the culture tanks [26].

Growth measurements followed standard procedures described by Sriphuthorn and Sanoamuang [9]. On each sampling day, ten shrimp were randomly collected from each replicate and measured individually. Body length was measured from the anterior margin of the head to the distal end of the telson using a digital Vernier caliper (precision: 0.01 mm), and wet body weight was measured using a digital analytical balance (readability: 0.01 g). Individual measurements were treated as subsamples, and replicate means were used for statistical analyses.

### 2.3. Feeding Regime

Fairy shrimp were fed twice daily at 08:00 and 16:00 throughout the experimental period. Feeding rates were adjusted according to developmental stage and observed feed consumption to ensure adequate feed availability while minimizing deterioration of water quality. During the early developmental stages, small feed portions were gradually provided to avoid feed accumulation, whereas larger quantities were supplied during later stages in accordance with increased biomass and feeding activity. Feeding management followed the protocols described by Saengphan et al. [8] and Sriputhorn and Sanoamuang [9].

For the 1–5-day group, shrimp in the control treatment were fed fresh *Chlorella* sp. at 1 × 10^6^ cells mL^−1^ day^−1^. The amount of dry feed provided to each treatment was adjusted to approximate the biomass equivalent of the control diet following the feeding protocols described by Saengphan et al. [8] and Sriputhorn and Sanoamuang [9]. Feeding quantities were increased for the 6–10 and 11–20-day groups according to shrimp growth and feed consumption.

### 2.4. Water Quality Measurement

Water quality parameters were measured at five-day intervals throughout the experimental period. On each sampling day, measurements were taken twice daily at 06:00 and 15:00 h. Parameters included pH, nitrite (mg L^−1^), ammonia (mg L^−1^), total alkalinity (mg L^−1^ as CaCO_3_), total hardness (mg L^−1^ as CaCO_3_), and dissolved oxygen (mg L^−1^). Measurements were conducted according to standard methods described by APHA [29].

### 2.5. Proximate Composition, Amino Acid, and Carotenoid Analyses

Moisture, crude protein, crude lipid, ash, and carbohydrate contents of fairy shrimp samples were analyzed using standard procedures of the Association of Official Analytical Chemists (AOAC) [30]. Crude protein was estimated from total nitrogen content using a conversion factor of 6.25.

Total carotenoid content was determined according to the method of Rodriguez-Amaya and Kimura [31] with slight modifications. Shrimp samples were homogenized and extracted with acetone under low-light conditions to minimize pigment degradation. Carotenoid extracts were quantified by high-performance liquid chromatography (HPLC), and the results were expressed as µg g^−1^ dry weight.

Amino acid profiles were determined after acid hydrolysis following AOAC procedures [30]. Prior to hydrolysis, samples were subjected to oxidation for sulfur-containing amino acid determination. Amino acid concentrations were subsequently analyzed using HPLC. Due to limited biomass availability, samples were pooled prior to biochemical analyses; therefore, proximate composition, carotenoid, and amino acid data are presented descriptively without statistical comparison.

### 2.6. Statistical Analysis

Data were analyzed using IBM SPSS Statistics for Windows, version 29.0.2.0 (20) (IBM Corp., Armonk, NY, USA). Normality and homogeneity of variances were assessed using the Shapiro–Wilk test and Levene’s test, respectively, prior to ANOVA. Differences in growth performance, survival, and water quality among treatments were analyzed using one-way analysis of variance (ANOVA). When significant differences were detected, means were separated using Duncan’s new multiple range test, which is commonly applied in aquaculture nutrition studies for comparing treatment means under controlled experimental conditions. Differences were considered statistically significant at *p* < 0.05. Individual shrimp measurements were treated as subsamples, and replicate means were used for analyses to avoid pseudoreplication.

## 3. Results

### 3.1. Growth Performance and Survival

Growth performance (body length and wet body weight) and survival rate of *S. sirindhornae* under the seven dietary treatments across three developmental stages (1–5, 6–10, and 11–20 days post-hatch) are summarized in Table 1.

During the early developmental stage (1–5 days post-hatch), body length was highest in FF1 and FF2 and significantly lower in FF7 (*p* < 0.05). Wet body weight was highest in FF1 and FF2 and significantly lower in FF5 and FF7 (*p* < 0.05). Survival rate followed a similar pattern, with FF1 and FF2 showing the highest survival rate and FF7 the lowest (*p* < 0.05).

During the intermediate developmental stage (6–10 days post-hatch), differences in growth performance among treatments became less pronounced compared with the early developmental stage. Body length was highest in FF2 and significantly lower in FF7 (*p* < 0.05). Wet body weight and survival rate did not differ significantly among treatments (*p* > 0.05).

As shrimp progressed into the last developmental stage (11–20 days post-hatch), differences in growth performance among treatments became minimal. No significant differences among treatments were observed for body length or wet body weight (*p* > 0.05). However, survival rate was significantly higher (*p* < 0.05) in FF1 and FF2 than in FF3–FF7.

Overall, FF2 consistently supported growth performance and survival rate comparable to those of the control diet (*Chlorella* sp.) across all developmental stages and generally performed better than the other dry diet formulations.

### 3.2. Proximate Composition, Carotenoids, and Amino Acid Profile

The proximate composition, carotenoid content, and amino acid profile of *S. sirindhornae* fed fresh *Chlorella* sp. (FF1) and the best-performing dried diet (FF2) are presented in Table 2. Shrimp fed FF2 showed higher protein, total lipid, carotenoid, ash, and energy contents than shrimp fed *Chlorella* sp., whereas moisture and total carbohydrate contents were lower.

FF2-fed shrimp also exhibited higher concentrations of both essential and non-essential amino acids. Lysine and leucine were the predominant essential amino acids in both treatments, with higher levels observed in FF2-fed shrimp. Similarly, glutamic acid and tyrosine were among the most abundant non-essential amino acids and were present at higher concentrations in FF2-fed shrimp. Arginine and hydroxylysine were detected at levels below the analytical detection limit in both treatments.

### 3.3. Water Quality

Mean (±SD) values of water quality parameters recorded during the culture of *S. sirindhornae* across the three developmental stages are summarized in Table 3. Water quality values represent pooled measurements across all dietary treatments and were analyzed according to developmental stage. All measured parameters, including pH, nitrite, ammonia, total alkalinity, total hardness, and dissolved oxygen, remained stable throughout the experimental period. Although relatively high variation was observed for some low-concentration parameters, particularly nitrite and ammonia, these fluctuations remained within acceptable ranges for fairy shrimp culture. No significant differences were observed among developmental stages for any parameter (*p* > 0.05). Overall, water quality conditions were consistent and remained within acceptable ranges under the applied feeding regime.

## 4. Discussion

The present study demonstrates that the FF2 diet, composed of equal proportions of spirulina powder and commercial complete shrimp feed, represents an effective algae-independent alternative to fresh *Chlorella* sp. for the culture of *S. sirindhornae*. Across all developmental stages examined, fairy shrimp fed FF2 consistently exhibited growth performance and survival rates comparable to those obtained with the control treatment and, where significant differences were observed, higher (*p* < 0.05) than those obtained with the poorest-performing diets, particularly FF6 and FF7. These findings indicate that the observed performance of FF2 was likely associated with its balanced nutritional composition and its ability to support adequate nutrient availability throughout development. However, although all diets were finely ground and passed through a 250-µm sieve prior to use, feed particle size distribution was not quantified. Therefore, the potential contribution of particle characteristics to the observed differences among dietary treatments cannot be completely excluded and warrants further investigation.

At the earliest developmental stage (1–5 days post-hatch), growth and survival of fairy shrimp fed FF2 were comparable to those fed fresh *Chlorella* sp., whereas reduced growth and survival were observed in shrimp fed diets lacking either spirulina or complete shrimp feed, particularly FF7. Early-stage fairy shrimp possess limited swimming ability and an incompletely developed digestive system, rendering feed particle size and nutrient density critical determinants of performance [8,26]. Although live microalgae remain well suited for early feeding due to their small particle size and suspension stability, the comparable performance of FF2 suggests that finely ground spirulina-based diets can provide adequate nutritional support during this sensitive ontogenetic stage when appropriately formulated. Nevertheless, the potential influence of feed particle size on the comparatively lower performance observed in some dried diet treatments cannot be excluded and should be further investigated in future studies. During the intermediate developmental stage (6–10 days post-hatch), dietary effects were most evident in body length, with shrimp fed FF2 exhibiting significantly greater growth than those fed FF6 and FF7, while body weight and survival did not differ significantly among treatments. This pattern suggests that dietary composition may initially influence linear body growth before resulting in detectable differences in biomass accumulation or survival. One possible explanation is that, during this transitional developmental stage, available nutrients may be preferentially allocated to body elongation and structural development, whereas measurable changes in body mass and survival may require a longer period of sustained nutrient intake. Such responses may also be influenced by differences in nutrient digestibility, feed particle accessibility, feed acceptance, or feeding efficiency among dietary treatments. Similar stage-dependent dietary responses have been reported in other fairy shrimp species cultured under controlled conditions [8,16].

In the final developmental stage (11–20 days post-hatch), survival emerged as the most sensitive indicator of dietary quality. Shrimp fed FF2 maintained survival rates equivalent to those fed fresh *Chlorella* sp. and significantly higher than those fed the other dried formulations. Survival during later developmental stages directly determines final biomass yield and production efficiency in nursery systems, highlighting the practical importance of FF2 as a substitute for live microalgae during extended culture periods. The comparable performance of FF2 may be associated with differences in feed acceptance, palatability, nutrient availability, or a combination of these factors. However, feed intake and nutrient utilization were not quantified in the present study; therefore, the mechanisms underlying the observed performance differences among diets remain speculative and warrant further investigation. Spirulina is widely recognized for its high protein content, favorable amino acid profile, and abundance of carotenoids, whereas commercial complete shrimp feed provides balanced levels of essential amino acids, dietary lipids, vitamins, and minerals required for crustacean growth and survival [12,32]. Therefore, the nutritional characteristics of these ingredients may have contributed to the observed performance of FF2. Nevertheless, the relative importance of feed acceptance, nutrient availability, and other dietary factors could not be determined from the present study and should be examined in future research.

This nutritional advantage was reflected in the biochemical composition of the shrimp biomass. As shown in Table 2, fairy shrimp fed FF2 exhibited higher protein, total lipid, and carotenoid contents on a dry-weight basis than shrimp fed fresh *Chlorella* sp. Proteins and lipids are fundamental macronutrients supporting tissue accretion and energy metabolism, while carotenoids contribute to antioxidant capacity and pigmentation in aquatic organisms [12,13]. These compositional characteristics reinforce the value of *S. sirindhornae* as a nutritionally rich live or processed feed for freshwater aquaculture species [14]. In addition, the amino acid profile of FF2-fed shrimp showed generally higher concentrations of both essential and non-essential amino acids than those of shrimp fed fresh *Chlorella* sp. Lysine and leucine were the most abundant essential amino acids detected in both treatments, with higher levels observed in FF2-fed shrimp. Lysine is frequently identified as a limiting amino acid in crustacean diets and plays a central role in protein synthesis and growth, while branched-chain amino acids such as leucine, isoleucine, and valine are closely associated with muscle protein accretion and energy metabolism [11,16]. However, these compositional differences may reflect differences in growth performance and physiological condition among treatments rather than direct causal determinants of improved culture performance. Because biochemical composition was assessed at the end of the feeding trial, the observed differences should be interpreted as being associated with, rather than necessarily responsible for, the superior performance observed in FF2-fed shrimp.

Water quality parameters remained stable throughout the experimental period and did not differ significantly among developmental stages, indicating that differences in growth and survival among dietary treatments were not confounded by environmental deterioration. Measured water quality parameters remained within acceptable ranges previously reported for fairy shrimp culture, including near-neutral to slightly alkaline pH values, dissolved oxygen concentrations above 5 mg L^−1^, low ammonia and nitrite concentrations, and moderate alkalinity levels under controlled rearing conditions [8,29]. This stability indicates that the feeding regimes applied in the present study did not result in excessive organic loading and were adequate for maintaining optimal culture conditions in controlled laboratory and nursery systems [29]. From a practical perspective, the FF2 formulation offers a viable algae-independent feeding strategy for fairy shrimp culture. The diet is composed of locally available and commercially accessible ingredients and can be readily applied under controlled laboratory or hatchery conditions. Although fresh *Chlorella* sp. remains suitable for early-stage feeding, reliance on live microalgae introduces operational challenges related to seasonal variability, labor requirements, and production consistency [8,23]. The present findings demonstrate that FF2 can support consistent culture performance across multiple developmental stages while reducing dependence on live algal production. Nevertheless, this study was conducted under controlled experimental conditions, and further research is warranted to evaluate the performance of FF2 under larger-scale production systems, such as concrete tanks or earthen ponds. Future studies incorporating economic evaluation, optimization of feed particle size for early developmental stages, and long-term reproductive performance would provide valuable insights into the commercial feasibility and sustainability of this feeding strategy.

Despite the promising results obtained with FF2, several limitations of the present study should be acknowledged. First, feed intake, palatability, and nutrient utilization were not directly quantified; therefore, the mechanisms underlying the observed differences in performance among dietary treatments could not be conclusively determined. Second, although all diets were finely ground and passed through a 250-µm sieve, particle size distribution was not measured, and its potential influence on feed ingestion cannot be completely excluded. Third, the study was conducted under controlled laboratory conditions and focused primarily on growth performance, survival, and biomass composition during the juvenile culture period. Consequently, the applicability of FF2 under commercial-scale production systems and its effects on reproductive performance remain to be evaluated. Future studies addressing these limitations, together with economic analyses and long-term production trials, would further strengthen the practical application of algae-independent feeding strategies for fairy shrimp culture.

## 5. Conclusions

This study demonstrates that FF2, a diet composed of equal proportions of spirulina powder and commercial shrimp feed, is an effective algae-independent alternative to fresh *Chlorella* sp. for the culture of the tropical fairy shrimp *S. sirindhornae*. Across all developmental stages, FF2 supported growth performance and survival rates comparable to those achieved with live microalgae, while producing shrimp biomass with enhanced protein, lipid, carotenoid, and amino acid contents. These results indicate that spirulina-based dried formulations can successfully replace live microalgae in the culture of *S. sirindhornae* without compromising growth performance or nutritional quality.

The proposed feeding strategy is particularly suitable for nursery-scale operations and for regions where fresh microalgal production is technically, seasonally, or economically constrained. However, this study was conducted under controlled experimental conditions, and detailed physicochemical characterization of all experimental diets, including particle size distribution and water stability, was beyond the scope of the present study. Further evaluation under large-scale production systems is therefore still required. Future studies focusing on economic feasibility, optimization of feed particle size during early developmental stages, long-term reproductive performance, and detailed feed characterization would provide valuable insights into the commercial applicability and sustainability of this feeding strategy.

Future prospects include the development of cost-effective algae-independent feeds for large-scale fairy shrimp production, refinement of nutritionally balanced formulations tailored to different developmental stages, and integration of such feeding strategies into freshwater aquaculture hatchery systems. These advances may contribute to more reliable fairy shrimp production, reduced dependence on live microalgae, and improved availability of high-quality live feed resources for freshwater aquaculture.

Overall, the findings provide a scientific basis for the development of practical and nutritionally balanced algae-independent feeding approaches for freshwater aquaculture hatchery systems.

## Figures and Tables

**Table 1:** Growth performance and survival of *S. sirindhornae* cultured under different dietary treatments across three developmental stages.

Developmental Stage	Feed Formula	Body Length (mm)	Wet Body Weight (mg)	Survival Rate (%)
1–5 days	FF1	9.80 ± 0.76 ^a^	0.40 ± 0.01 ^a^	66.90 ± 2.89 ^a^
	FF2	9.85 ± 1.24 ^a^	0.40 ± 0.02 ^a^	63.60 ± 2.74 ^a^
	FF3	9.42 ± 0.51 ^ab^	0.30 ± 0.08 ^bc^	45.90 ± 12.60 ^ab^
	FF4	9.36 ± 0.20 ^ab^	0.30 ± 0.11 ^bc^	45.33 ± 11.52 ^ab^
	FF5	9.29 ± 0.46 ^ab^	0.20 ± 0.03 ^c^	37.20 ± 29.00 ^bc^
	FF6	9.44 ± 0.25 ^ab^	0.30 ± 0.04 ^ab^	37.00 ± 20.10 ^bc^
	FF7	8.24 ± 0.25 ^b^	0.20 ± 0.03 ^c^	30.96 ± 12.00 ^c^
6–10 days	FF1	12.54 ± 0.31 ^abc^	19.6 ± 3.9 ^a^	65.75 ± 5.25 ^a^
	FF2	14.06 ± 1.22 ^a^	23.0 ± 5.2 ^a^	69.55 ± 9.35 ^a^
	FF3	13.06 ± 0.75 ^abc^	19.4 ± 4.7 ^a^	69.10 ± 4.50 ^a^
	FF4	13.46 ± 1.01 ^ab^	16.7 ± 12.6 ^a^	68.45 ± 13.45 ^a^
	FF5	12.73 ± 0.61 ^abc^	24.1 ± 5.6 ^a^	65.75 ± 15.85 ^a^
	FF6	11.93 ± 1.33 ^bc^	15.5 ± 12.0 ^a^	67.15 ± 15.85 ^a^
	FF7	11.46 ± 0.98 ^c^	19.8 ± 1.1 ^a^	62.65 ± 3.25 ^a^
11–20 days	FF1	18.40 ± 1.38 ^a^	87.0 ± 14.4 ^a^	61.85 ± 4.25 ^a^
	FF2	18.53 ± 0.70 ^a^	87.5 ± 8.1 ^a^	62.45 ± 5.28 ^a^
	FF3	18.20 ± 0.80 ^a^	86.0 ± 6.5 ^a^	44.45 ± 4.55 ^b^
	FF4	17.83 ± 0.94 ^a^	80.3 ± 1.1 ^a^	42.25 ± 2.75 ^b^
	FF5	17.60 ± 1.11 ^a^	67.0 ± 13.0 ^a^	40.65 ± 4.15 ^b^
	FF6	17.93 ± 0.11 ^a^	77.7 ± 5.6 ^a^	41.78 ± 6.95 ^b^
	FF7	17.66 ± 0.64 ^a^	73.3 ± 13.6 ^a^	38.66 ± 5.53 ^b^

Footnote: Values are presented as mean ± SD (*n* = 3 replicate containers per treatment). Within each developmental stage, different superscript letters within a column indicate significant differences among dietary treatments (*p* < 0.05; one-way ANOVA followed by Duncan’s new multiple range test).

**Table 2:** Proximate composition, carotenoid content, and amino acid composition of *S. sirindhornae* fed fresh *Chlorella* sp. and FF2.

Component	*S. sirindhornae*		Unit
	Fed with *Chlorella* sp.	Fed with FF2	
Proximate composition			
Ash	0.47	0.54	g/100 g
Energy	17.21	24.29	kcal/100 g
Moisture	95.64	94.10	g/100 g
Protein (N × 6.25)	3.18	4.73	g/100 g
Total carbohydrate	0.38	0.06	g/100 g
Total lipid	0.33	0.57	g/100 g
Carotenoids	25.20	37.79	µg g^−1^ dry weight
**Amino acid composition**			
**Essential amino acids (EAA)**			
Histidine	182.32	372.36	mg/100 g
Isoleucine	199.32	262.89	mg/100 g
Leucine	372.58	545.88	mg/100 g
Lysine	679.02	1144.00	mg/100 g
Methionine	60.11	85.31	mg/100 g
Phenylalanine	333.86	445.94	mg/100 g
Threonine	24.09	52.23	mg/100 g
Tryptophan	26.55	55.23	mg/100 g
Valine	137.82	181.33	mg/100 g
**Total EAA**	**2015.67**	**3145.17**	mg/100 g
**Non-essential amino acids (N-EAA)**			
Alanine	109.21	137.74	mg/100 g
Aspartic acid	70.87	166.40	mg/100 g
Cystine	26.43	61.65	mg/100 g
Glutamic acid	251.09	454.37	mg/100 g
Glycine	71.50	95.02	mg/100 g
Proline	79.56	107.38	mg/100 g
Serine	13.29	33.03	mg/100 g
Tyrosine	233.19	368.48	mg/100 g
**Total N-EAA**	**855.14**	**1424.07**	mg/100 g
**EAA:N-EAA ratio**	**2.36**	**2.21**	—
**Other/conditionally essential amino acids**			
Arginine	<5.00	<0.50	mg/100 g
Hydroxylysine	<0.50	<0.50	mg/100 g
Hydroxyproline	9.11	10.19	mg/100 g

Footnote: Values are expressed on a dry-weight basis. Carotenoid content is expressed as µg g^−1^ dry weight. Total EAA and total N-EAA were calculated from detected amino acids. Amino acids below the detection limit, as well as modified or conditionally essential amino acids, were excluded from the EAA:N-EAA ratio calculation. Values below the detection limit are indicated as “<”. Values are presented as descriptive analytical data obtained from pooled samples; therefore, no statistical comparisons were performed.

**Table 3:** Mean (±SD) water quality parameters recorded during the culture of *Streptocephalus sirindhornae* in 5-L circular plastic containers across three developmental stages (1–5, 6–10, and 11–20 days post-hatch).

Water Quality Parameters	1–5 Days	6–10 Days	11–20 Days
pH	7.500 ± 1.013 ^a^	7.100 ± 0.489 ^a^	6.920 ± 0.493 ^a^
Nitrite (mg L^−1^)	0.160 ± 0.183 ^a^	0.100 ± 0.194 ^a^	0.110 ± 0.144 ^a^
Ammonia (mg L^−1^)	0.540 ± 0.271 ^a^	0.330 ± 0.188 ^a^	0.340 ± 0.320 ^a^
Alkalinity (mg L^−1^ as CaCO_3_)	44.000 ± 11.73 ^a^	44.000 ± 14.29 ^a^	34.000 ± 9.660 ^a^
Hardness (mg L^−1^ as CaCO_3_)	60.000 ± 21.080 ^a^	65.000 ± 24.150 ^a^	55.000 ± 15.810 ^a^
Dissolved oxygen (mg L^−1^)	9.900 ± 1.370 ^a^	10.100 ± 1.646 ^a^	11.500 ± 3.415 ^a^

Footnote: Values are presented as mean ± SD. Within each row, means sharing the same superscript letter are not significantly different among developmental stages (*p* > 0.05). Water quality data were pooled across all dietary treatments (FF1–FF7) and analyzed only among developmental stages (1–5, 6–10, and 11–20 days post-hatch). Water quality parameters were monitored at 5-day intervals and measured twice daily (06:00 and 15:00 h) according to APHA [29]. The parameters presented in this table were evaluated as indicators of overall culture conditions and were not compared among dietary treatments.

## Data Availability

Data supporting the findings of this study are available from the corresponding author upon reasonable request.
